# Root enhancement in cytokinin-deficient oilseed rape causes leaf mineral enrichment, increases the chlorophyll concentration under nutrient limitation and enhances the phytoremediation capacity

**DOI:** 10.1186/s12870-019-1657-6

**Published:** 2019-02-20

**Authors:** Erika Nehnevajova, Eswarayya Ramireddy, Andrea Stolz, Maria Gerdemann-Knörck, Ondřej Novák, Miroslav Strnad, Thomas Schmülling

**Affiliations:** 1 Institute of Biology/Applied Genetics, Dahlem Centre of Plant Sciences (DCPS), Freie Universität Berlin, Albrecht-Thaer-Weg 6, 14195 Berlin, Germany; 2 Czech Academy of Sciences, Institute of Experimental Botany & Palacký University, Olomouc, Czech Republic; 3 Present address: Indian Institute of Science Education and Research (IISER) Tirupati, Biology Division, 517507, Tirupati, Andhra Pradesh India

**Keywords:** *Brassica napus*, Cytokinin, Cytokinin oxidase/dehydrogenase, Leaf mineral enrichment, Phytoremediation, Plant growth, Root enhancement

## Abstract

**Background:**

Cytokinin is a negative regulator of root growth, and a reduction of the cytokinin content or signalling causes the formation a larger root system in model plants, improves their growth under drought and nutrient limitation and causes increased accumulation of elements in the shoot. Roots are an important but understudied target of plant breeding. Here we have therefore explored whether root enhancement by lowering the cytokinin content can also be achieved in oilseed rape (*Brassica napus* L.) plants.

**Results:**

Transgenic plants overexpressing the *CKX2* gene of *Arabidopsis thaliana* encoding a cytokinin-degrading cytokinin oxidase/dehydrogenase showed higher CKX activity and a strongly reduced cytokinin content. Cytokinin deficiency led to the formation of a larger root system under different growth conditions, which was mainly due to an increased number of lateral and adventitious roots. In contrast, shoot growth was comparable to wild type, which caused an enhanced root-to-shoot ratio. Transgenic plants accumulated in their leaves higher concentrations of macro- and microelements including P, Ca, Mg, S, Zn, Cu, Mo and Mn. They formed more chlorophyll under Mg- and S-deficiency and accumulated a larger amount of Cd and Zn from contaminated medium and soil.

**Conclusions:**

These findings demonstrate the usefulness of ectopic *CKX* gene expression to achieve root enhancement in oilseed rape and underpin the functional relevance of a larger root system. Furthermore, the lack of major developmental consequences on shoot growth in cytokinin-deficient oilseed rape indicates species-specific differences of *CKX* gene and/or cytokinin action.

**Electronic supplementary material:**

The online version of this article (10.1186/s12870-019-1657-6) contains supplementary material, which is available to authorized users.

## Background

Cytokinins are key regulators of numerous developmental and physiological processes [1–5]. Essential steps of their metabolism and signal transduction have been elucidated in *Arabidopsis thaliana* [reviewed by [6, 7]]. Breakdown of cytokinins is catalyzed by the seven members of the cytokinin oxidase/dehydrogenase (CKX) family. *CKX* genes and proteins differ in their expression profiles, subcellular localisations and biochemical characteristics [8, 9].

Constitutive overexpression of *CKX* genes in tobacco and *Arabidopsis* plants resulted in plants with reduced cytokinin content showing a compound phenotype called the cytokinin deficiency syndrome [10, 11]. Plants showing this syndrome are characterized by slow-growing, stunted shoots with small leaves and an enhanced root system. Consistently, mutants of cytokinin receptor genes and other mutants of cytokinin metabolism and signalling genes show similar phenotypic changes [12–18]. Essentially, this and other work [19–22] has established cytokinin as a negative regulator of root growth and branching.

Interestingly, limiting the expression of *CKX* genes mainly to roots of transgenic tobacco (*Nicotiana tabacum*), *Arabidopsis* or barley (*Hordeum vurlgare*) plants enabled the production of plants with a larger root system lacking the otherwise detrimental consequences of cytokinin deficiency for the shoot [23–26]. These plants were shown to be more resistant to drought stress, form more chlorophyll under Mg- and S-limitation and to accumulate higher concentrations of several elements in their leaves and grains. Thus targeted expression of *CKX* genes is a promising tool to engineer plants with an enhanced root system and to explore its potential benefits.

Recently roots have come into focus for improvement of crop plants, and it has been argued that the relevance of the root system as a breeding target in crop plants has been underestimated [27–31]. Analysis of various crop plants with a modified root system architecture obtained by genetic engineering revealed that an enhanced root system can be advantageous under several circumstances [reviewed in [32]]. For instance, in rice overexpression of the *PHOSPHORUS-STARVATION TOLERANCE1* (*PSTOL1*) gene caused increased root biomass and enhanced grain yield by more than 60% under phosphate-deprived conditions [33]. Expression of the *DEEPER ROOTING1* (*DRO1*) gene in a shallow-rooting rice variety (IR64) caused an increase in root growth angle, which resulted in a steep-deep root architecture enabling the plants to achieve higher yields under drought conditions [34].

Here we have tested whether the function of cytokinin as a negative regulator of root development also holds true in oilseed rape (*Brassica napus* L.), which is an important oil-producing crop plant closely related to Arabidopsis. *B. napus* (2n = 38, AACC) has a recently (~ 7500 years ago) formed allotetraploid genome resulting from a hybridization event of the *B. rapa* (2n = 20, AA) and *B. oleracea* (2n = 18, CC) genomes [35]. Based on the comparative genomic analysis, it was estimated that approximately 90.3 and 71.4% of the genomic components of the *B. rapa* and *B. oleracea* genomes were conserved in *B. napus* [36].

In the genome of *B. napus* a large number of cytokinin metabolism and signalling genes was identified. Among others, there are 26 cytokinin-synthesizing *IPT* and 23 *CKX* genes*,* which are differentially expressed [37, 38] as compared to nine *IPT* and seven *CKX* genes in Arabidopsis. The fact that a large number of cytokinin genes have been retained in the *Brassica* genome suggests that the hormone exerts numerous distinct functions in this species. In addition, mapping studies in *B. rapa* and *B. napus* revealed that members of the *CKX* gene family and cytokinin signalling genes are linked to yield-related loci which are of potential use for breeding in *Brassica* species [36, 39–41]. Consistently, certain combinations of *CKX* gene mutations have led to a yield increase in Arabidopsis [42]. Here we show that the overexpression of the *Arabidopsis CKX2* gene in oilseed rape causes root enhancement in the absence of a strong impact on shoot development. The cytokinin-deficient plants accumulate higher concentrations of different elements in their leaves, form more chlorophyll under Mg- and S-deficiency and have an improved phytoremediation capacity. Thus, the concept of root enhancement by ectopic *CKX* gene expression is extended to an important crop species.

## Results

### Generation of cytokinin-deficient oilseed rape plants

In order to study the consequences of a constitutively reduced cytokinin content in oilseed rape, we attempted to obtain plants expressing two prototypic *CKX* genes of *Arabidopsis thaliana*, *CKX1* and *CKX2*, under the control of the 35S promoter [10]. Overexpression of both genes causes strongly enhanced root growth in the model plants tobacco and Arabidopsis. However, *CKX1* overexpression leads to a dramatic reduction of shoot growth, while *CKX2* overexpression causes only a moderate reduction of shoot growth [10, 11].

**Fig. 1:**
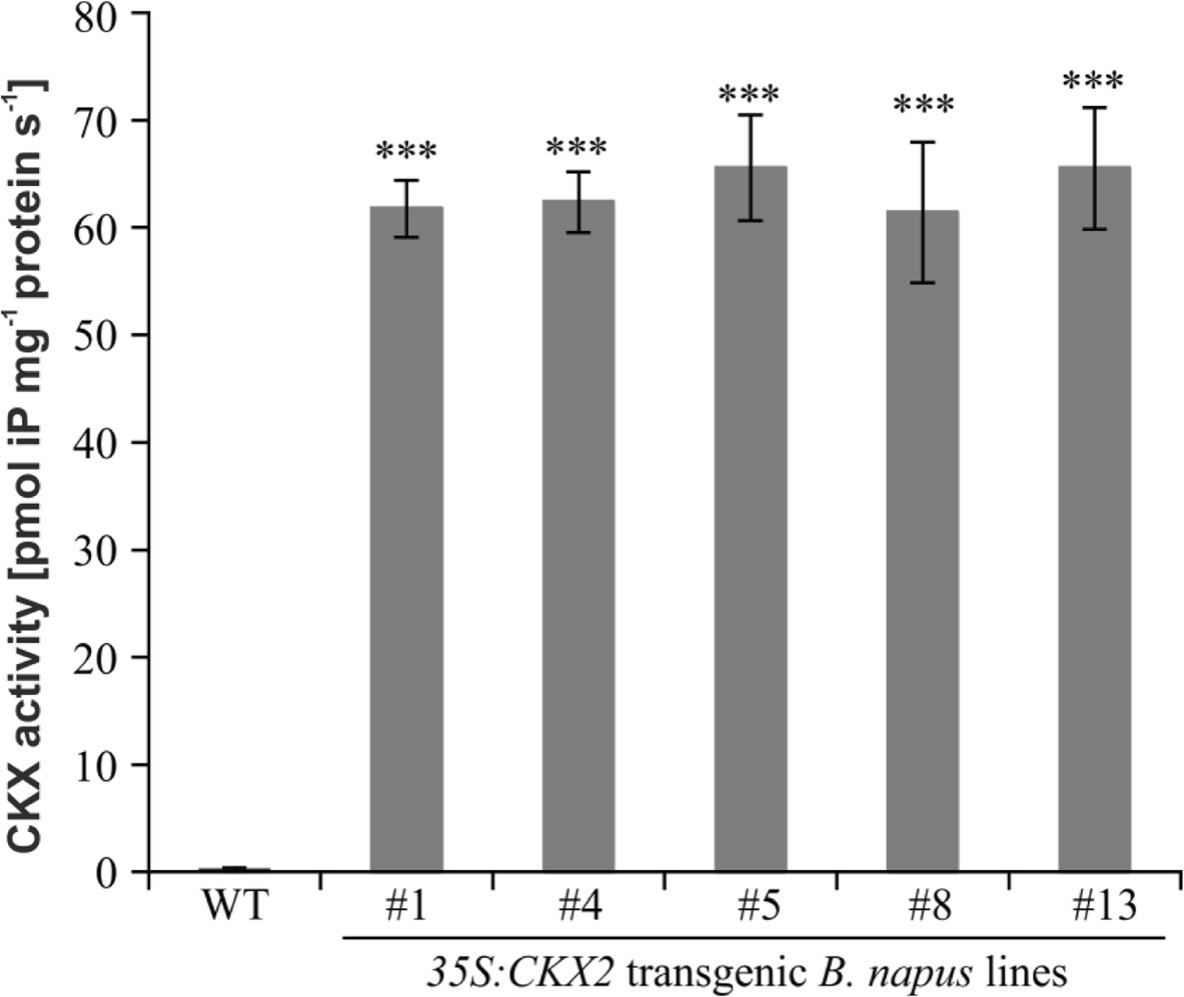
*35S:CKX2* transgenic *B. napus* plants displayed increased cytokinin oxidase/dehydrogenase enzyme (CKX) activity. For each genotype three independent biological replicates of three pooled 8-day-old seedlings cultivated in vitro were analysed. Data represent mean values ± SD. Student’s *t*-test was used to calculate the significance of differences between transgenic lines and wild type. *** *p* < 0.001

Attempts to obtain *35S:CKX1* transgenic plants of oilseed rape failed repeatedly indicating that the gene product activity interferes strongly with the regeneration process which requires cytokinin. In contrast, thirteen independent transformants displaying a similar phenotype were obtained for the *35S:CKX2* gene. CKX activity was tested in seedlings of five of these lines and all showed a strongly enhanced CKX activity (> 60 pmol iP mg^− 1^ protein s^− 1^) as compared to wild-type seedlings (0.3 ± 0.08 pmol iP mg^− 1^ protein s^− 1^) (Fig. 1). Further characterization was carried out with homozygote progeny obtained by self-fertilization from three independent lines, *35S:CKX2–1*, *35S:CKX2–4* and *35S:CKX2–13*.

Analysis of the cytokinin concentration in root, shoots or whole 8-d-old seedlings of these transgenic lines revealed a low (below 1 pmol g FW^− 1^) cytokinin concentration in wild-type seedlings, which is about one order of magnitude lower than in Arabidopsis. In the transgenic lines, the concentration of different cytokinin metabolites was reduced to a similar extent (Table 1 and Additional file 1: Table S1). Among the iP-type cytokinins, the only significant and consistent change was a reduction of isopentenyl-9-*N*-glucoside (iP9G) in roots to 14–23% of the wild-type concentration. All other iP-type metabolites were below the detection limit or not significantly altered. The concentration of the biologically active *trans*-zeatin (*t*Z), was in most tissues and transgenic lines lowered to about 30–50% of the concentration in wild type. Similar reductions were noted for *t*Z riboside, glucoside and monophosphate (Table 1). In contrast, the concentration of several *cis*-zeatin (*c*Z)-type cytokinins (*c*Z and *cis*-zeatin 9-glucoside, *c*Z9G) was not altered significantly in the *35S:CKX2* lines. An exception is a 2–4.5-fold increase of *c*Z5´-RMP in roots of all three transgenic lines. This unexpected increase may reflect a homeostatic mechanism that activates the synthesis of *c*Z-type cytokinin in response to the lowered concentration of iP-and *t*Z-type cytokinins. Similar changes and cytokinin metabolite differences were measured in 14-d-old seedlings (Additional file 1: Table S1). Taken together, overexpression of the *CKX2* gene caused a strong increase of CKX activity and a significant decrease of the cytokinin content.

**Table 1:** Cytokinin content of 8-d-old *Brassica napus* seedlings overexpressing the *CKX2* gene. The concentration of cytokinin metabolites was measured in root, shoot and whole seedlings of the lines *35S:CKX2-1*, *35S:CKX2-4*, *35S:CKX2-13*, and wild type. The cytokinin metabolite concentrations detected in the transgenic lines are shown as percent of those found in wild type. Absolute values and statistical analysis are shown in Additional file 1: Table S1. Percentage printed in bold indicates statistically significant differences (*p* < 0.05) as compared to wild type calculated by Student’s *t*-test

	*35S:CKX2-1*			*35S:CKX2-4*			*35S:CKX2-13*		
	Root	Shoot	Seedling	Root	Shoot	Seedling	Root	Shoot	Seedling
iP-type									
iP	nd	188	**42**	nd	173	**42**	247	nd	52
iPR	nd	nd	nd	nd	83	279	80	180	192
iP9G	**23**	nd	**45**	**20**	nd	nd	**14**	nd	**40**
iPR5′MP	162	89	58	100	160	128	101	179	94
*t*Z-type									
*t*Z	**31**	nd	58	**31**	nd	140	**37**	**22**	**45**
*t*ZR	**50**	**42**	**32**	**83**	**35**	**45**	79	**59**	**31**
*t*Z9G	69	nd	10	**28**	nd	28	**27**	nd	7
*t*ZOG	na	**33**	40	na	**28**	27	na	nd	nd
*t*ZR5′MP	78	63	**22**	108	**22**	**23**	76	**21**	**29**
*c*Z-type									
*c*Z	**38**	63	**51**	**30**	**33**	**57**	**26**	**35**	**55**
*c*ZR	116	91	**59**	109	94	87	92	105	99
*c*Z9G	90	93	124	**76**	86	**159**	**79**	82	98
*c*ZOG	**60**	nd	90	71	nd	nd	153	nd	nd
*c*ZR5′MP	**290**	57	96	**191**	94	173	**475**	107	106

*Abbreviations*: *tZ trans*-zeatin, *tZR t*Z riboside, *tZ9G t*Z 9-*N*-glucoside, *tZOG t*Z *O*-glucoside, *tZR5′MP t*ZR 5′-monophosphate, *tZROG t*ZR *O*-glucoside, *cZ cis*-zeatin, *cZR c*Z riboside, *cZ9G c*Z 9-*N*-glucoside, *cZOG c*Z *O*-glucoside, *cZR5′MP c*ZR 5′-mono phosphate, *iP* N6-(Δ2-isopentenyl)adenine, *iPR* iP riboside, *iP9G* iP 9-*N*-glucoside, *iPR5′MP* iPR 5′-monophosphate, *nd* concentration below the detection limit, *na* not applicable due to a concentration below detection limit in wild type

### Cytokinin-deficiency causes enhanced root growth

**Fig. 2:**
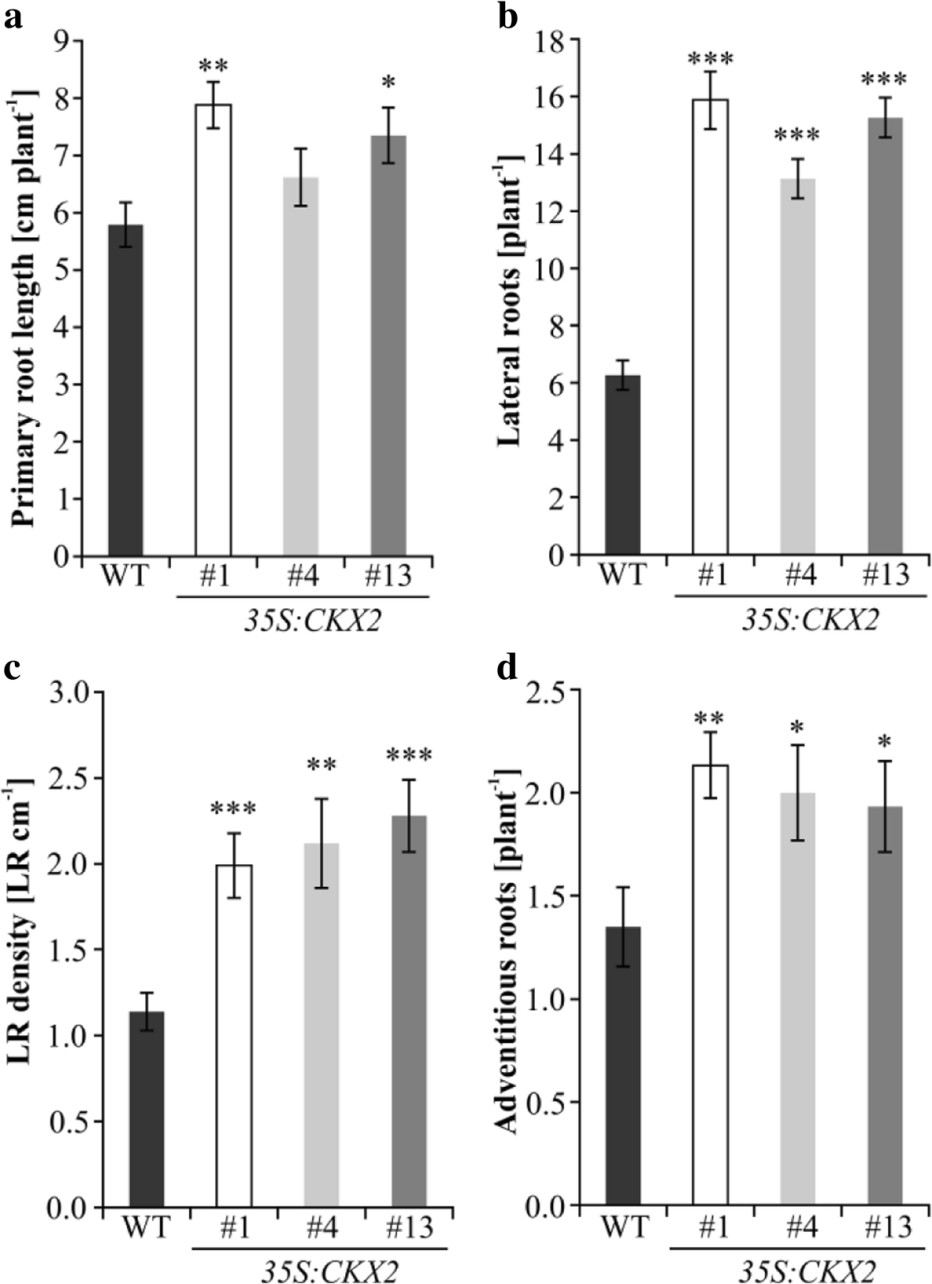
*35S:CKX2* transgenic *B. napus* lines have an increased root system. **a** Primary root length, (**b**) number of lateral roots, (**c**) lateral root density, and (**d**) number of adventitious roots of seedlings (6 DAG) cultured in vitro on vertical plates on half strength MS medium. Data represent mean values ± SEM (*n* ≥ 15). Two-tailed Student’s *t*-test was performed to calculate the significance of differences between transgenic lines and wild type. * *p* < 0.05;** *p* < 0.01;*** *p* < 0.001

Next, we investigated whether the altered cytokinin content influences the root or shoot development of oilseed rape. The root architecture was analysed from plants grown under different growth conditions, namely in vitro, in a hydroponic system or in soil. The length of the primary root of 6-day-old seedlings grown in vitro differed between wild type and the transgenic lines (Fig. 2 a). Two of the transgenic lines (*35S:CKX2–*1 and *35S:CKX2–*13) showed a significant 25–35% increase of their primary root length compared to wild type (Fig. 2a). About twice as many lateral roots (LR) were formed in all three transgenic lines compared to wild type (Fig. 2 b). This increase in LR number resulted in a strong increase of LR density (70–100%) in the transgenic lines (Fig. 2 c, d). Furthermore, under in vitro growth conditions the number of adventitious roots was also increased by about 40–50% in the transgenic plants compared to wild type (Fig. 2 d).

**Fig. 3:**
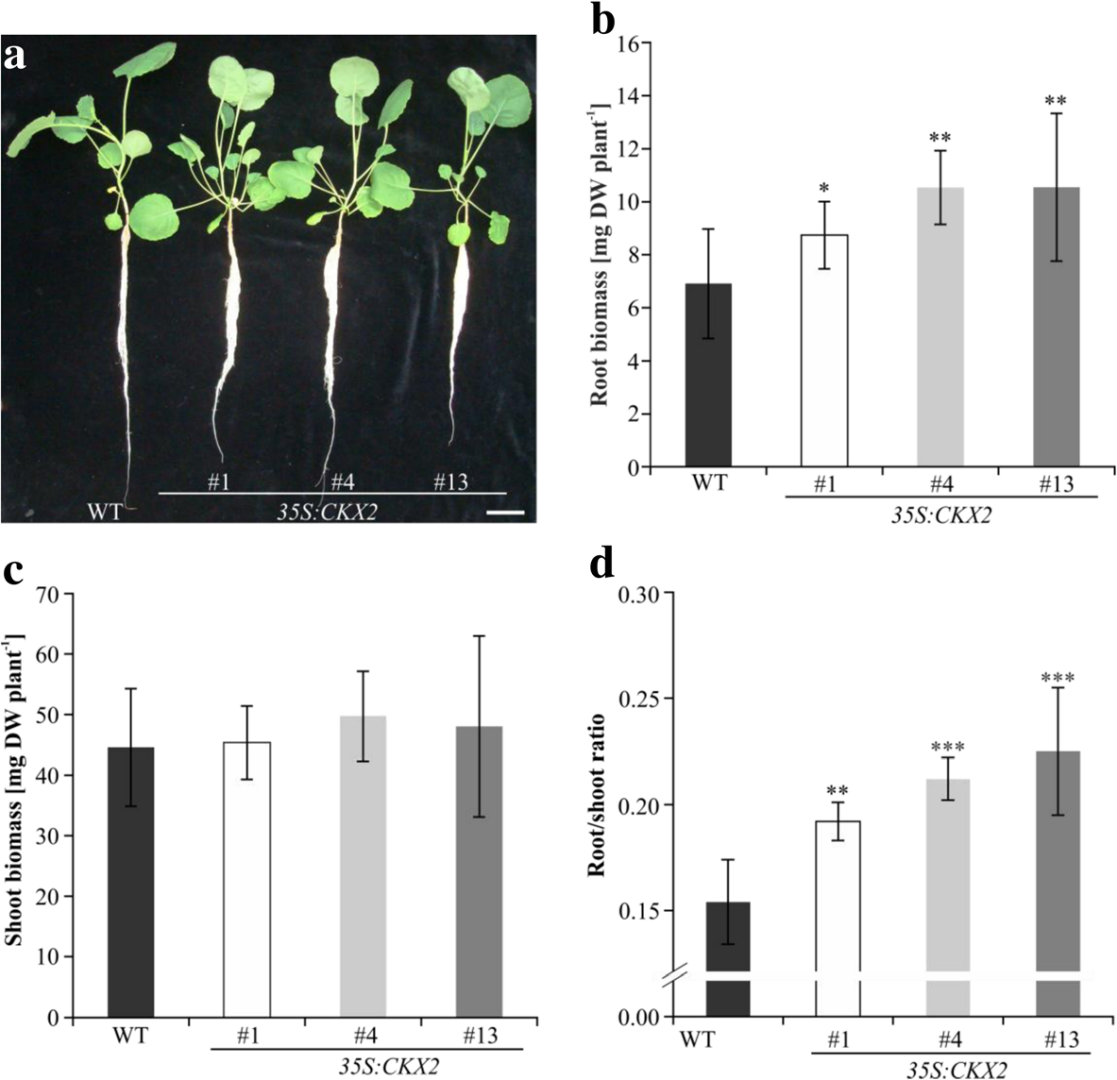
Phenotype of *35S:CKX2* transgenic *B. napus* plants grown in a hydroponic system. **a** Growth habit of five-week-old *35S:CKX2* transgenic *B. napus* lines. Scale bar = 1 cm. **b** Root and (**c**) shoot dry mass of three independent *35S:CKX2* transgenic *B. napus* lines. Data represent mean values ± SD. *n* = 10 (**d**) Root-shoot ratio is increased by up to 46% in transgenic plants. Significance of differences between transgenic lines and wild type were calculated using Student’s *t*-test. * *p* < 0.05; ** *p* < 0.01; *** *p* < 0.001

We then analysed the root system size of plants grown in a hydroponic system (Fig. 3 a). Visual inspection of five-week-old plants indicated that the primary root lengths of transgenic lines were not increased under these conditions. However, the root mass of transgenic lines was more voluminous suggesting that their number of primary and secondary lateral roots was increased compared to wild type (Fig. 3 a). The dry weight of roots of *35S:CKX2* lines was increased up to 50% in comparison to wild type (Fig. 3 b). In contrast, the shoot dry mass was comparable between wild type and the transgenic lines (Fig. 3 c). This differential growth increased the root-to-shoot biomass ratio by 25–46% in the transgenic lines compared to wild-type plants (Fig. 3 d).

**Fig. 4:**
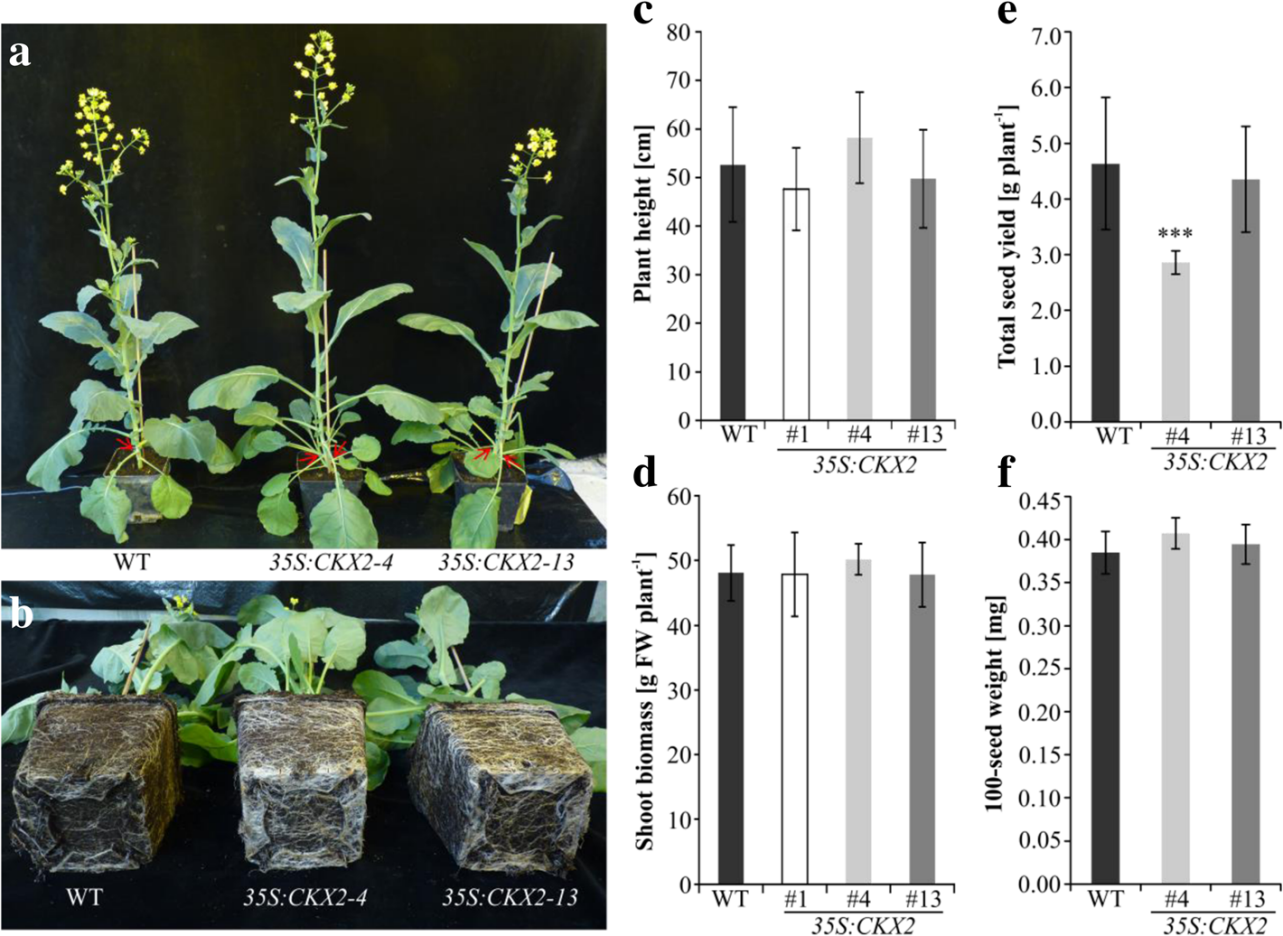
Phenotype of 10-week-old soil-grown *35S:CKX2* transgenic *B. napus* lines. **a** Growth habit of plants. Lateral buds in the leaf axils of transgenic plants developed two to three small leaves during early vegetative development (indicated by red arrows). (**b**) Enhanced root system of *35S:CKX2* transgenic plants compared to wild type. (**c**) Plant height of transgenic lines and wild type at the onset of flowering. (*n* = 20). **d** Fresh weight of *35S:CKX2* transgenic shoots compared to wild type. (*n* = 10). (e) Total seed yield, and (f) 100-seed weight of transgenic plants compared to wild type. (*n* = 15–18). Data were obtained from soil-grown plants after 8 weeks (c) or 12 weeks (d) of cultivation in a greenhouse. Data in (c-f) represent mean values ± SD. The statistical significance of differences compared to wild type was calculated using two-tailed Student’s *t-*test. ***, *p* < 0.001

Visual inspection of soil-grown plants also confirmed the formation of a larger root system in the transgenic lines compared to wild type (Fig. 4 a, b). In contrast, generally only very minor developmental changes were noted in the shoots of the transgenic lines. 84 DAG plant height (Fig. 4 a, c) and shoot fresh weight (Fig. 4 d) of transgenic plants was similar compared to wild type. However, during vegetative development the lateral buds in the leaf axils of transgenic plants produced two to three small leaves, in contrast to the lateral buds of wild type, which remained completely inhibited (Fig. 4 a). Further, total yield (Fig. 4 e) and 100-seed weight (Fig. 4 f) of two lines were compared to wild-type plants. In one of the lines the total seed yield was similar to wild type while a reduction of of 38% was found in the second line. The 100-seed weight was unchanged in both transgenic lines. Taken together, *35S:CKX2* transgenic *B. napus* plants formed an increased root system but showed generally no major anomalies of their shoot development.

### *CKX* transgenic plants accumulate higher element concentrations in their leaves

**Fig. 5:**
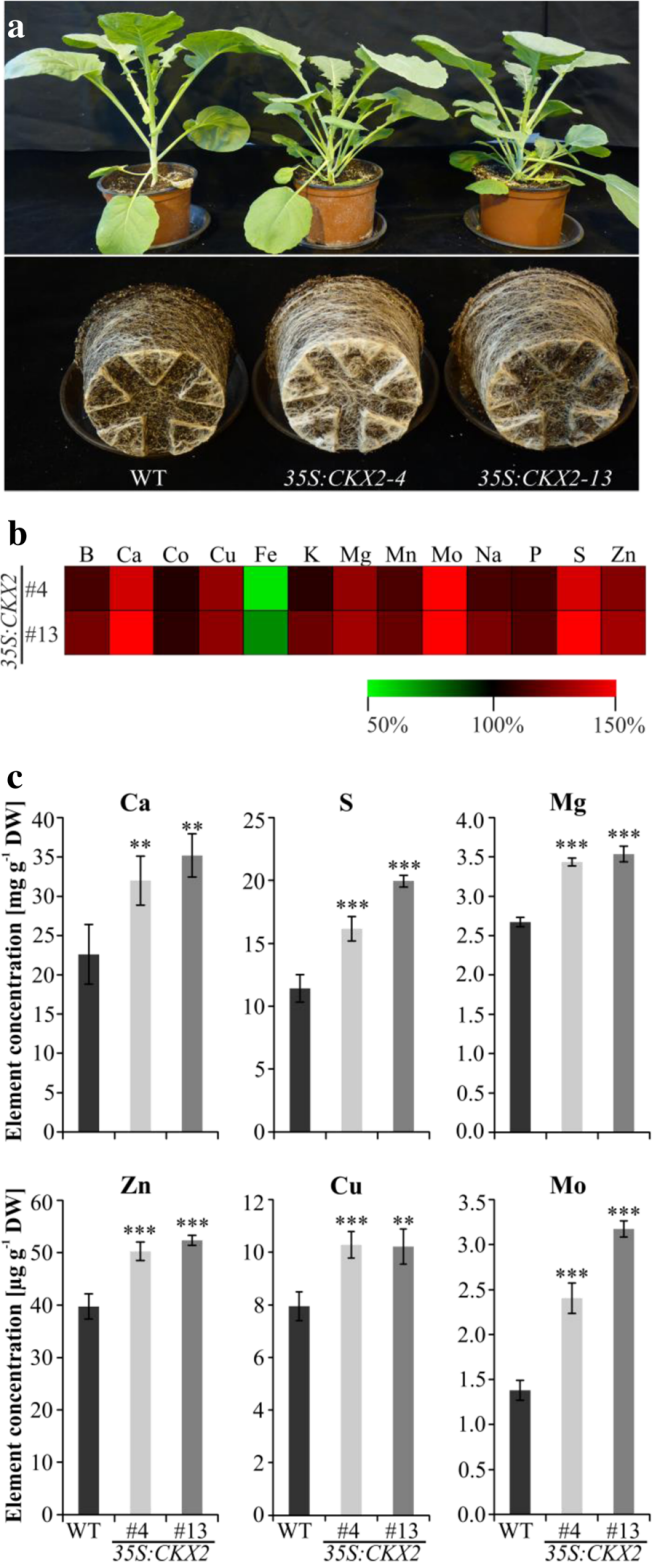
Transgenic *B. napus* plants have an enhanced mineral element concentration in their leaves. **a** Growth habit of one-month-old *35S:CKX2* transgenic *B. napus* lines grown on unfertilized soils supplemented with nutrient solution. **b** Heat map showing that *35S:CKX2* transgenic plants accumulate higher element concentrations as wild type. Mineral element concentration in wild type was set to 100%. The heat map was generated using Multiexperiment Viewer v4.9. **c** Concentration of selected macro- and microelements in leaves of wild type (WT) and transgenic lines. Quantification of mineral elements was done in one-month-old soil-grown plants as described in Materials and methods. Four biological replicates for each genotype were analyzed. Each biological replicate contained leaves from two plants. Data shown in (**c**) represent mean values ± SD. The statistical significance of differences compared to wild type was calculated using two-tailed Student’s *t-*test (* *p* < 0.05; **, *p* < 0.01; ***, *p* < 0.001)

Previous work with *CKX* transgenic *Arabidopsis* and barley plants has shown that root enhancement may cause the accumulation of enhanced concentrations of different elements in leaves [23, 25]. In order to analyse whether this is also the case in *CKX* transgenic oilseed rape, we measured the leaf element concentration in one-month-old plants. Similar to Arabidopsis, the transgenic oilseed rape lines accumulated higher concentrations than wild type of most of the quantified elements that are quantified when compared to wild-type plants (Fig. 5 and Additional file 2: Table S2). For example, all the five macroelements that were quantified were present in higher concentration in transgenic plants. These included phosphorus (plus 13 and 16% in the two lines), calcium (plus 41 and 56%), sulfur (plus 42 and 75%), and magnesium (plus 29 and 32%) (Fig. 5 b, c and Additional file 2: Table S2). Similarly, the concentration of the essential microelements zinc (plus 26 and 32%), copper (plus 29 and 28%), molybdenum (plus 74 and 130%) and manganese (15 and 20%) was also significantly increased in these lines (Fig. 5 b and c). Only the concentration of iron was reduced by 18 and 11% in these lines as compared to wild type (Fig. 5b and Additional file 2: Table S2). These results indicate that also in oilseed rape plants an increased root system caused by cytokinin-deficiency enhances the uptake of mineral elements from the soil and their transport to the above-ground part.

### The chlorophyll concentration of *CKX* transgenic plants is enhanced under Mg- and S-deficiency

**Fig. 6:**
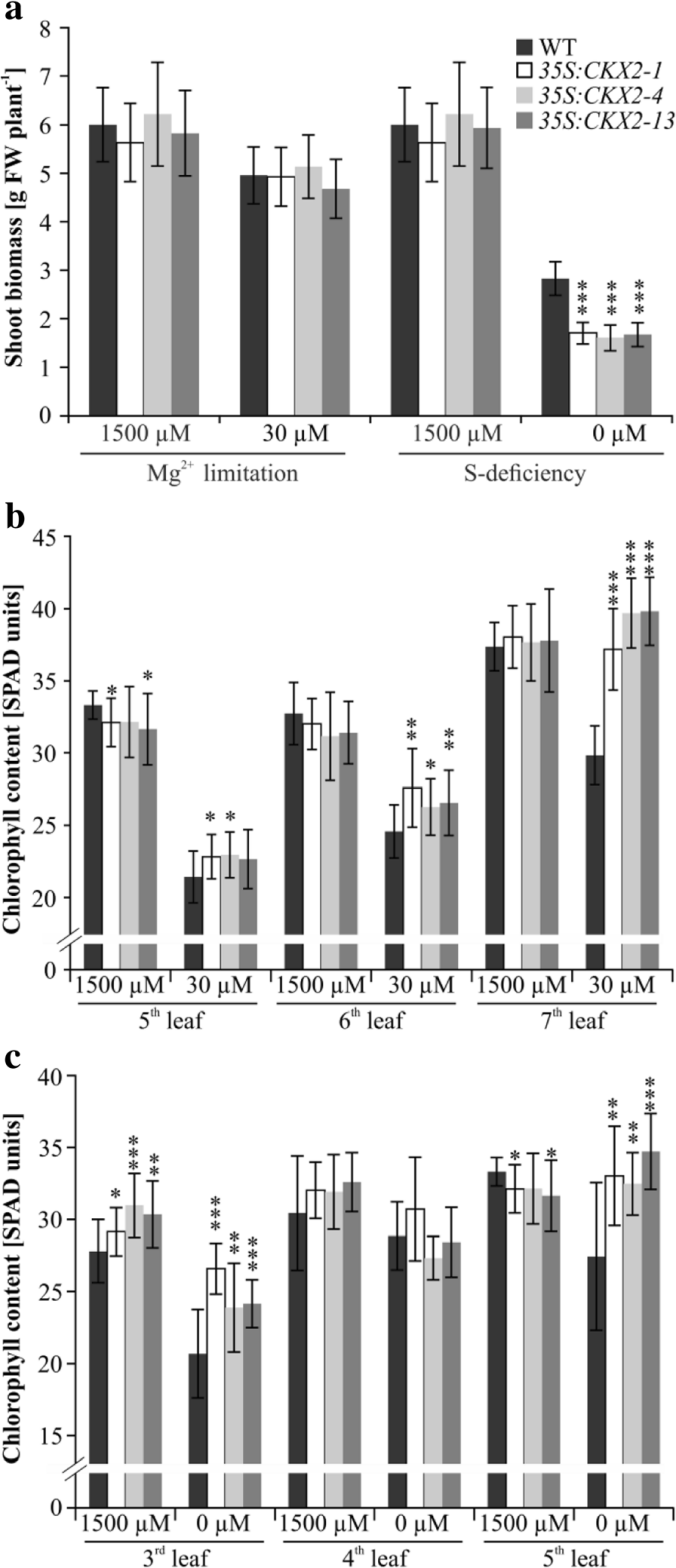
Shoot growth and leaf chlorophyll content of *35S:CKX2* transgenic *B. napus* lines grown under Mg^2+^ and SO_4_^2−^ limitation. **a** Shoot biomass (fresh weight) of transgenic plants grown on a perlite substrate supplemented with Hoagland nutrient solution containing different concentrations of Mg^2+^ and SO_4_^2−^. **b**, **c** Leaf chlorophyll concentration of plants grown under Mg^2+^ (**b**) or SO_4_^2−^ (**c**) limitation. SPAD readings were taken on plants that were exposed to the indicated nutrients regimes for 3 weeks and shoot fresh weight was determined after 5 weeks. Data in (**a**), (**b**) and (**c**) represent mean values ± SD (*n* = 18). Student’s *t*-test was used to compare the significance of differences between transgenic lines and the wild type. * *p* < 0.05; ** *p* < 0.01; *** *p* < 0.001

Next, we studied whether the increased root system of transgenic *B. napus* lines has any physiological relevance under nutrient-limiting conditions. It is known that cytokinin influences biosynthesis and concentration of chlorophyll as well as photosynthesis [43, 44]. Further, it was shown for tobacco that an enhanced root system resulting from cytokinin- deficiency might be advantageous for growth under Mg- and S-limiting conditions [23]. Being the central element in chlorophyll, Mg is an essential component of the photosynthetic apparatus [45]. In case of sulphur (S), *Brassicaceae* are known as high S-demanding plants compared to other crop plants [46]. Oilseed rape is particularly sensitive to S-deficiency, which reduces both seed quality [47] and yield [48]. Both Mg- and S-deficiency cause a strong decrease in leaf chlorophyll concentration [49, 50]. Therefore, the leaf chlorophyll concentration of transgenic *B. napus* and wild-type plants grown under suboptimal concentrations of Mg or S were compared (Fig. 6).

Under Mg-limiting conditions, shoot fresh weight of wild-type and transgenic plants was reduced to a similar extent (about 18–20%) compared to standard conditions (Fig. 6 a). Under S-deficiency, shoot growth was strongly reduced in both genotypes but the shoot weight of transgenic plants was even lowered by about 20% more than in wild-type plants (i.e. reduced by 70–72% in the former and by 53% in the latter) (Fig. 6 a). The symptoms of Mg-limitation such as interveinal chlorosis on older lower leaves with a marbling effect were shown by both genotypes (data not shown). Quantification of the chlorophyll concentration of older leaves (leaf 5, 6 and 7) revealed that with saturating Mg concentrations (1500 μM) in the medium the chlorophyll concentration was similar in different leaves of wild type and the transgenic lines with a somewhat lower concentration in the 5th leaf of the latter (Fig. 6 b). Under Mg-limitation (30 μM) all leaves of transgenic lines showed a significantly higher chlorophyll concentration than leaves of wild type. This difference was larger in the oldest leaf (7th leaf; ca. 25–33% more chlorophyll) than in younger leaves (Fig. 6 b).

Under S-deficient conditions (0 μM S) plants of both genotypes showed diffuse yellowing of their youngest leaves (data not shown). Transgenic plants showed a significantly higher chlorophyll concentration in the youngest leaves (3rd and 5th leaf, up to 25% more chlorophyll) compared to wild type (Fig. 6 c). Together, these results are consistent with previous results obtained with *CKX* transgenic tobacco plants [23].

### Cytokinin-deficient plants accumulate higher amounts of cd and Zn from contaminated substrates

**Fig. 7:**
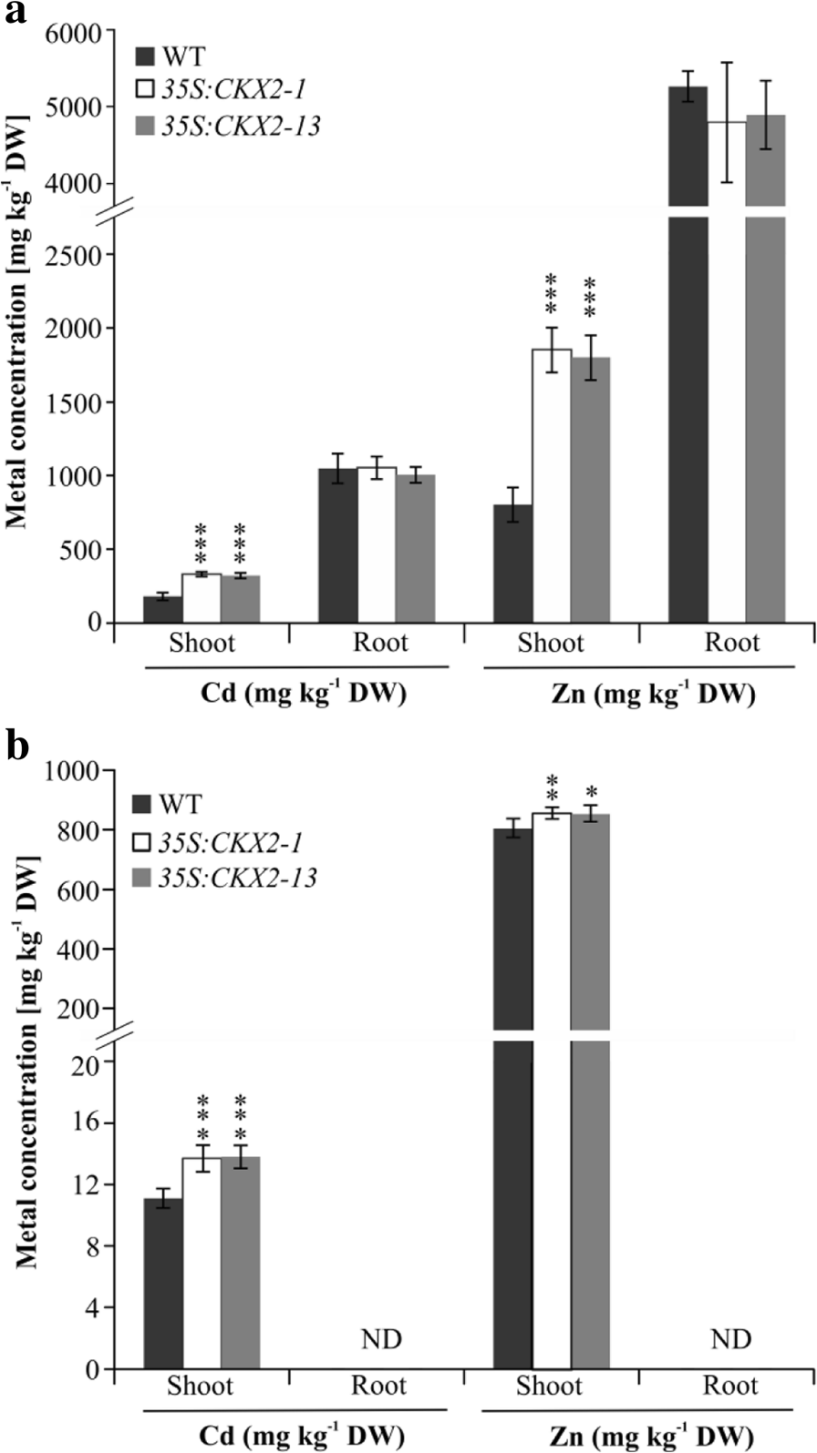
Concentration of Cd and Zn in shoots and roots of *35S:CKX2* transgenic *B. napus* plants grown on contaminated substrate. **a** Metal concentrations in shoots and roots of three-week-old plants grown in a hydroponic culture supplemented with 5 μM Cd or 50 μM Zn. **b** Metal concentrations in shoots and roots of six-week-old plants grown in soil containing 10 mg kg^− 1^ Cd and 1100 mg kg^− 1^ Zn. Data represent mean values ± SD (*n* = 9–10). Student’s *t*-test was used to calculate the significance of differences in metal concentration between transgenic lines and wild type. * *p* < 0.05; ** *p* < 0.01; *** *p* < 0.001; ND, not determined

Plants with a highly branched root system accessing a large soil volume are ideal candidates for phytoremediation strategies. It has been shown before that an extensive root system of plants may lead to an enhanced uptake of heavy metals or increased rhizodegradation of xenobiotics from contaminated soil [51–54]. To explore whether the larger root system of *CKX* transgenic oilseed rape provides in this respect any advantage compared to the near-isogenic wild type, we compared growth and accumulation of the heavy metals cadmium (Cd) and zinc (Zn) in wild-type and transgenic plants. Plants were grown either in a hydroponic system containing medium supplemented with 5 μM Cd and 50 μM Zn or in soil containing 10 mg kg^− 1^ Cd and 1100 mg kg^− 1^ Zn. The shoot biomass of the transgenic lines and wild type were similar both in standard and metal-containing medium or soil (data not shown). Transgenic *B. napus* plants grown in the hydroponic system accumulated up to 80 and 120% more Cd and Zn in their shoots compared to wild-type plants (Fig. 7 a). Also, soil-grown *CKX* transgenic plants accumulated higher concentration of these elements, however the difference to wild type was lower, i.e. 25% for Cd and 6% for Zn (Fig. 7 b).

## Discussion

This work has shown that ectopic expression of *CKX* genes might be used to construct oilseed rape plants with a larger root system, reported the phenotypic changes this causes and revealed a number of interesting facts about the action of cytokinin in this species.

We extended the concept of achieving root enhancement through ectopic expression of a *CKX* gene, which had been shown previously in model plants [23–26]. Curiously, the *35S:CKX2* plants studied here originated from a pilot study aiming to test different prototypic *CKX* genes in *B. napus*. The finding that *CKX2* overexpressing oilseed rape show only very minor phenotypic effects in the shoot was unexpected as *35S:CKX2* transgenic tobacco and *Arabidopsis* plants do show a reduced shoot growth although less strongly than plants expressing *35S:CKX1* [10, 11]. Another difference compared to these other species has been that several attempts to generate transgenic oilseed rape plants expressing *35S:CKX1* failed, which indicates that its expression strongly interferes with shoot formation during the generation of transgenic plants [see [55–58] for the negative impact of CKX activity on plant regeneration]. The CKX1 and CKX2 proteins show different subcellular localisations and biochemical characteristics. A tagged CKX1 protein was detected predominantly in the endoplasmic reticulum [59] while a CKX2-GFP fusion protein was located in the ER but moved possibly also to the apoplastic space [11, 60]; for review see [61]. The CKX1 protein preferred cytokinin ribosides and *N*^9^-glucosides as substrates, while iP and iPR where preferred by CKX2 [9]. Together this suggests that these two CKX proteins degrade distinct cellular cytokinin pools. The data reported here indicate that the cytokinin pool accessible to CKX2 does not play an important role for shoot development in *B. napus*, while it is relevant to regulate root system architecture and size. In contrast, the cytokinin pool accessible to CKX1 is apparently important for plant regeneration and presumably also for later shoot development. In addition, it could be that cytokinin activities have different threshold values in roots and shoots, which is indicated by the higher sensitivity of roots in cytokinin bioassays as compared to shoots. Finally, the larger root system may provide more favourable conditions for shoot growth, thus partly compensating for the negative effect of cytokinin deficiency. Notably, transgenic expression of a cytokinin synthesis gene in oilseed rape altered shoot traits [62, 63] documenting the responsiveness of the oilseed rape shoot to the hormone. The distinct action of different cytokinin pools and CKX enzymes is relevant to design future approaches to modulate the cytokinin status in oilseed rape.

Another interesting finding has been that the total cytokinin content of *B. napus* seedlings is about one order of magnitude lower than in Arabidopsis (see [11, 23, 64, 65] for comparison) but the relative reduction in cytokinin content achieved in both species by systemic *CKX2* expression appears to be similar. For example, the *t*ZR and *t*ZRMP concentration was about twenty times higher in seedlings of *Arabidopsis thaliana* but also lowered to about 30% of the wild-type level in *35S:CKX2* transgenic seedlings [11, 23, 64]. Another notable difference concerns the relative abundance of *N*- and *O*-glucosides in *B. napus* and *Arabidopsis* seedlings. For example, *t*Z9G was below the detection level in *B. napus*, whereas in *Arabidopsis* it was present in high concentration [23, 64, 65]. In contrast, the concentration of *c*Z9G and *c*ZOG was similar (i.e. in the range of 1 pmol g^− 1^ FW) in *Arabidopsis* and oilseed rapeseed seedlings and the concentration was in both species not lowered by overexpression of *CKX2*. These data document that the cytokinin concentration and metabolite profile even of relatively closely related species can differ significantly. It will be interesting to study whether this is reflected by changes in cytokinin sensitivity and output of the signaling system.

As a consequence of cytokinin-deficiency, the root system of transgenic *B. napus* lines was increased significantly compared to wild type under all tested growth conditions (Fig. 2, Fig. 3 and Fig. 4). This increase was mainly attributed to the strongly increased formation of lateral roots, which had almost doubled. The about two-fold reduction of the distance between newly formed lateral roots was larger than in tobacco and Arabidopsis where it is in the range of 30–50% [22, 23, 66]. One might conclude that cytokinin acts as a positional cue in lateral root formation in *B. napus* as it does in *Arabidopsis* [66]. Consistently, the *B. napus lrn1* mutant, which is insensitive to exogenous cytokinins forms also more lateral roots compared to wild type [67]. Further, *CKX2* transgenic *B. napus* plants showed an increased formation of adventitious roots similar to other species with a reduced cytokinin status [11, 14, 68, 69]. However, in contrast to the strong effect of cytokinin deficiency on lateral root and adventitious root formation, the impact of cytokinin deficiency on elongation of the primary root was only weak [10, 11, 23]. This is consistent with different dose-response curves for the impact of cytokinin on different root traits. For example, also in Arabidopsis primary root elongation is less sensitive to changes in cytokinin than lateral root formation [22, 66].

An interesting similarity between Arabidopsis and oilseed rape plants with an increased root system was the increased concentration of the same mineral elements (P, Ca, Mg, S, Zn, Cu and Mo) in their leaves [23]. Likewise, the leaf iron concentration was decreased by 30% in Arabidopsis [23] and by 11–18% in oilseed rape (Fig. 5). It is conceivable that the increased root system of the transgenic lines explores a larger soil volume and thus has access to a larger nutrient reservoir. However, as the concentration of elements is not always changed in the same way or to a similar extent, there must be other factors influencing the observed changes. It has been shown that altered expression of specific transporter genes as a consequence of the lowered cytokinin concentration might contribute to a more efficient (or in case of Fe less efficient) uptake of elements from the soil and/or their transport to the above-ground parts. For Arabidopsis, an increased expression of genes encoding sulfate, phosphate, Mn, and Zn transporter was reported for cytokinin-deficient genotypes [23]. Furthermore, recent work has revealed that cytokinin regulates the formation of passage cells in the root endodermis to enable uptake across an otherwise impermeable barrier [70]. It could be that an altered passage cell number in cytokinin-deficient roots is one more cause for the enhanced element concentrations in the shoots of these plants.

Leaves of transgenic *Brassica* plants were enriched with important dietary elements (Ca, Mg, Zn) indicating that root enhancement has a potential to biofortify plants of the genus *Brassica* and other crop plants. The lack of nutrients such as Ca, Mg and Zn in the diet can cause serious health problems and increasing the concentration of elements in crop plants is an explicit goal of breeding efforts [71, 72]. Leaves of *Brassica* crops are important for human nutrition as this genus includes vegetables such as *Brassica rapa* (Chinese cabbage, pak choi and turnip) and *Brassica oleracea* (broccoli, cabbage and cauliflower)*.* Thus, efforts have been made to biofortify these plants with mineral elements that are often lacking in human diets [72]. For example, in *Brassica oleracea* QTLs associated with shoot Ca and Mg concentration were identified in order to breed *Brassica* plants with higher shoot Ca and Mg concentration to improve human dietary intakes [73].

The higher concentration of Mg and S in leaves of *35S:CKX2* transgenic *B. napus* plants might be the reason why these plants showed a significantly higher chlorophyll concentration (up to 25–30% more chlorophyll) in leaves under Mg- and S-limitation conditions (Fig. 6 b and c). This is interesting as soils deficient in mineral nutrients are one of the main reasons for poor crop yield and seed quality. Particularly S-deficiency has been recognized as a constraint to sustainable crop production in many parts of the world including Europe [74]. Oilseed rape being a high S-demanding crop is sensitive to S-deficiency. S-deficiency in oilseed rape leads to reduced growth, leaves become chlorotic and show reduced photosynthetic activity [49]. It was shown in *B. juncea* and *B. campestris* that high S-fertilization increases the levels of Rubisco, chlorophyll, and total protein content in fully expanded upper leaves, which implies a better photosynthetic activity in comparison with plants grown without S [50]. Together it suggests that increase of chlorophyll concentration is a way for coping with S-deficiency in the soil and sustain better growth. However, this was not the case in *35S:CKX2* transgenic plants under the growth conditions studied here, which indicates that other factors became limiting for shoot growth. For example, it could be that the reduced cytokinin concentration in shoots becomes a limiting factor. Indeed, transgenic tobacco plants with a root-specific reduction of cytokinin also formed more chlorophyll under Mg- and S-limitation but lacked the growth depression noted for *35S:CKX2 B. napus* under these conditions [23].

Phytoremediation is considered as the cheapest and sustainable technology for cleaning up contaminated soil. In case of heavy metal contamination, phytoextraction in which plants accumulate heavy metals in their above-ground organs is regarded as most suitable technique. In recent years, *Brassica* species have been used for phytoextraction because of their inherent capability to hyperaccumulate metals [75, 76]. It has been shown before that cytokinin deficiency mediated increase of root system size can accumulate higher concentrations of mineral elements efficiently in their above-ground parts [23]. In the present study we showed that *35S:CKX2* transgenic *B. napus* plants accumulate up to two-fold more Cd and Zn in their shoots when grown in hydroponics or in a contaminated soil (Fig. 7) suggesting that use of plants with an enhanced root system might be an option to improve phytoremediation strategies. Importanly, Cd and other heavy metals do not accumulate to high concentrations in the edible oil of oil crops including oil seed rape [77]. Moreover, the oil obtained from *B. napus* plants grown on contaminated soils can be used as a source of biodiesel production [78].

## Conclusions

In sum, the consequences of the overexpression of the *CKX2* gene in oilseed rape have demonstrated the function for cytokinin as a negative regulator of root growth in this species. This, together with the finding that the consequences of *CKX* gene expression can be limited mainly to the root [23, 27, 79] underpins the potential of a targeted decrease of the root cytokinin to achieve an improved performance of crop plants under nutrient or drought stress conditions. These results are of interest as in recent years the role of the root system for plant productivity has gained more importance. It has been argued that root-related traits have been understudied and should obtain more attention in order to face the future global need of increased food production [29, 32]. Several studies using transgenic approaches to modify root traits including root growth showed that this can be beneficial for plant health and stress resistance [32–34, 80–84]. The ectopic expression of *CKX* genes provides one additional option for altering the root system in a targeted fashion.

## Methods

### Plant material and growth conditions

*Brassica napus* L. cv. Kristina was used in this study. Homozygous *35S:CKX2–1*, *35S:CKX2–4* and *35S:CKX2–13* lines representing independent transformants were used in all experiments. Plants were cultured in vitro on MS medium under 16-h-light/8-h-dark cycles at 20 °C or were grown in a glasshouse with16-h-light/8-h-dark cycles at 22 °C and 18 °C.

### Oilseed rape transformation

The *35S:CKX1* and *35S:CKX2* genes combined with a hygromycin resistance gene as selectable marker were described previously [10]. Oilseed rape seeds were surface-sterilized with a mixture of 1.2% sodium hypochlorite and 1% Triton X-100 for 15 min and then with 70% ethanol for 1 min. The seeds were washed twice with sterile distilled water and germinated on agar-solidified half-strength MS medium [85]. Hypocotyl explants were excised from 7-d-old seedlings, cut into 5–7 mm long segments and transformed using *Agrobacterium tumefaciens* strain GV3101 harboring *35S:CKX1* or *35S:CKX2* as described by De Block et al. [86].

### Determination of CKX enzyme activity

Oilseed rape seedlings were cultured for 8 days in vitro on half strength MS medium solidified with 1% phytagel. Three seedlings were pooled and powdered in liquid nitrogen using a hand mortar. Three replicates each consisting of three pooled seedlings were analyzed for each line. The extraction method for the CKX2 enzyme and spectrophotometric measurement of enzyme activity was performed according to Galuszka et al. [9].

### Analysis of the cytokinin concentration

Cytokinin concentrations were measured in seedlings cultivated under the same in vitro conditions and harvested in the same way as plants used for analysis of CKX enzyme activity. 100–200 mg of roots and shoots were pooled separately for each sample and three independent biological replicates were analysed for each genotype and tissue. Extraction, purification and quantification of cytokinins by ultra-performance liquid chromatography-electrospray tandem mass spectrometry was performed according to the method described by Novák et al. [87], including modifications described in Novák et al. [88].

### In vitro root growth assay

Oilseed rape seeds were germinated on half-strength MS medium solidified with 1% phytagel in vitro on vertically positioned plates. The length of primary root and number of emerged lateral and adventitious roots was scored under a stereomicroscope at six days after germination (DAG).

### Morphometric analyses of plants grown in hydroponic culture

Seeds of three transgenic lines (*35S:CKX2–1; − 4; − 13*) and wild type were germinated and cultivated for three weeks in vitro on G1 medium [89] in WECK® glass vessels. Afterwards, primary root length and formation of lateral roots was assessed and seedlings were inserted in perforated plastic plates placed on 10 l vessels (ten seedlings per genotype in each vessel) filled with 9 l of 1/10-strength Hoagland medium modified as described [90]: 0.4 mM Ca(NO3)_2_; 0.2 mM MgSO_4_; 0.1 mM KH_2_PO_4_; 0.5 mM KNO_3_; 0.01 mM Na(FeEDTA); 10.01 μM H_3_BO_3_; 2 μM MnSO_4_; 0.2 μM CuSO_4_; 0.2 μM ZnSO_4_; 0.1 μM Na_2_MoO_4_; 20 μM NaCl. MES (2-(N-morpholino) ethanesulphonic acid) buffer was added to a final concentration of 2 mM and the solution was adjusted to pH 6.0 with 1 M KOH. The pH was measured at regular intervals during the experiment and did not vary more than 0.2 pH units. The nutrient solution was replaced twice a week. After 14 days, shoots and roots were harvested separately and their dry mass determined. To expose oilseed rape lines to Cd and Zn, 5 μM CdCl_2_ or 50 μM Zn (as ZnSO_4_) was added after one week of cultivation in standard 1/10 Hoagland medium. Plants were cultured for two further weeks, Hoagland solution containing Cd or Zn was replaced twice a week.

### Morphometric analyses of soil-grown plants

Transgenic *Brassica* lines and wild type were grown in pots in a greenhouse for either 8 or 12 weeks. Quantitative growth parameters were obtained from ten individuals of three independent transgenic clones and wild type. To explore accumulation of heavy metals, ten seedlings of each line were cultivated for six weeks on a sewage sludge contaminated soil. Sewage sludge (pH 6.3) was kindly provided by BioPlanta GmbH (Leipzig, Germany) from the sludge deposit Schladitz (Germany) and contained 10 mg kg^− 1^ Cd and 1110 mg kg^− 1^ Zn.

To measure the influence of different Mg concentrations on leaf chlorophyll concentration seeds were germinated in pots filled with perlite (Knauf Perlite GmbH, Dortmund, Germany) and grown in 1/10-strength modified Hoagland nutrient solution in the greenhouse. After two weeks plantlets (9 plants per genotype and treatment) were transferred to new pots filled with perlite and modified nutrient solution and grown for three further weeks. Control plants were fed with 1/10-strength Hoagland solution (1500 μM MgSO_4_), whereas plants cultivated under Mg deficiency were grown in a solution, which contained sub-optimal Mg-concentration (30 μM MgSO_4_) or S-deficiency (0 μM MgSO_4_). During the Mg-limitation study, MgSO_4_ in the medium was replaced by K_2_SO_4_ in order to maintain a constant anion/cation balance to avoid sulphur deficiency. In the S-deficiency study MgSO_4_ was compensated with an equivalent concentration of MgCl_2_ to avoid Mg-deficiency.

### Analysis of leaf element concentration

Seeds of two independent transgenic lines (35S:CKX2–4 and 35S-CKX2–13) and wild type were germinated on filter paper in vitro. Three DAG, seedlings were transferred to the greenhouse into an unfertilized (type-0) soil supplied by the company Einheitserde (Sinntal-Altengronau, Germany). Composition of unfertilized soil was tested and certified by Institut Koldingen GmbH (Sarstedt, Germany) as described by Drechsler et al. [91]. Plants were grown further for four weeks by supplementing equal amounts of fertilizer solution every two or three days depending on soil moisture. The fertilizer solution was based on the composition of 0.5x MS medium containing 10 mM KNO_3_ 10 mM NaH_2_PO_4_, 1 mM MgSO_4_, 1 mM CaCl_2_, 50 μM Na-Fe-EDTA, 50 μM H_3_BO_3_, 50 μM MnSO_4_, 18.5 μM ZnSO_4_, 50 nM CuSO_4_, 50 nM CoCl_2_, 0.5 μM NaMoO_4_ and 2 mM MES. The solution was adjusted to pH 5.7 with 1 M KOH. Leaf samples from one-month-old plants were dried for 72 h at 65 °C, equal amount were weighed into polytetrafluoroethylene tubes and digested with a HNO_3_ + H_2_O_2_ mixture in a pressurized microwave digestion system (UltraCLAVE IV from MLS GmbH, Leutkirch, Germany). The concentrations of macro- and microelements were analyzed by inductively-coupled plasma optical emission spectrometry (ICP-OES iCAP 6500 dual OES spectrometer, Thermo Fischer Scientific, Waltham, U.S.A.) with certified standards reference materials as control.

### Analysis of the cadmium and zinc concentrations in plants grown on contaminated substrate

After two weeks of growth in Cd- or Zn-containing medium or six weeks on sewage sludge contaminated soil plants were harvested and separated into shoots and roots. The plant material was dried at 80 °C for 48 h until a constant dry mass (DM) was reached. Samples for metal analyses were ground by an ultra-centrifugal mill (Retsch ZM 1) and passed through a 500 μm stainless mesh screen. Samples (250 mg DM) were digested with a HNO_3_ + H_2_O_2_ mixture by using a microwave digestion unit (MLS 1200 Mega). The concentrations of metals (Cd and Zn) were determined by flame atomic absorption spectrometry (FAAS) (Perkin Elmer 1100 B) as described [92, 93]. Certified reference material (Cabbage BCR-679) and two in-house standards from the European Environment Institute (Ispra, Italy) (mallow TP-29, tobacco TP-27; (https://ec.europa.eu/jrc/en/reference-materials) were included in each set of measurement for quality control.

### Chlorophyll measurement

Chlorophyll was determined by a SPAD-502 chlorophyll meter (Spectrum Technologies, Inc. Plainfield, Illinois, USA) after three weeks of growth under Mg- or S-deficiency. Two independent readings were taken in the middle part of each indicated leaf blade of nine individual plants per genotype, avoiding to place the chlorophyll meter over major leaf veins.

## Additional files

Additional file 1: **Table S1.** Cytokinin concentration in *Brassica napus* seedlings overexpressing the *CKX2* gene. The table represents the complete data set, which is supplementary to Table 1. The table shows the mean cytokinin content in 1 g of extracted tissue in pmol g^− 1^ F.W. ± SD (*n* = 3). Significance of differences compared to wild type (WT) was calculated using Student’s *t*-test (* *p* ≤ 0.05; ** *p* ≤ 0.01; *** *p* ≤ 0.001). LOD, limit of detection. NA, not applicable because two out of three biological replicates of this genotype have values below LOD. (DOC 175 kb)

Additional file 2: **Table S2.** Leaf element concentration in leaves of one-month-old *Brassica napus* plants overexpressing the *CKX2* gene. The table represents the complete data set, which is supplementary to Fig. 5. The table shows the mean element content in 1 g of extracted tissue in mg g^− 1^ DW or μg g^− 1^ DW ± SD (*n* = 4 for each data point). Each biological replicates contains pooled leaf samples of two independent plants. Data were compared using Student’s *t*-test (* *p* ≤ 0.05; ** *p* ≤ 0.01; *** *p* ≤ 0.001). DW, dry weight. (DOC 46 kb)

## Abbreviations

Cd: Cadmium
CKX: Cytokinin oxidase/dehydrogenase
*c*Z: *Cis*-zeatin
iP: N6-(Δ2-isopentenyl)adenine
LR: Lateral roots
Mg: Magnesium
S: Sulfur
*t*Z: *Trans*-zeatin
Zn: Zinc
