# Supplementary material for: Root enhancement in cytokinin-deficient oilseed rape causes leaf mineral enrichment, increases the chlorophyll concentration under nutrient limitation and enhances the phytoremediation capacity
Source: BMC Plant Biol. 2019 Feb 20;19:83. doi: 10.1186/s12870-019-1657-6 (PMC6381662; doi:10.1186/s12870-019-1657-6)
Supplement: Supplementary file 1 — Table S1. Cytokinin concentration in Brassica napus seedlings overexpressing the CKX2 gene. The table represents the complete data set, which is supplementary to Table 1. The table shows the mean cytokinin content in 1 g of extracted tissue in pmol g− 1 F.W. ± SD (n = 3). Significance of differences compared to wild type (WT) was calculated using Student’s t-test (* p ≤ 0.05; ** p ≤ 0.01; *** p ≤ 0.001). LOD, limit of detection. NA, not applicable because two out of three biological replicates of this genotype have values below LOD. (DOC 175 kb) [file 12870_2019_1657_MOESM1_ESM.doc]

**Table S1** **Cytokinin content in *Brassica napus* seedlings overexpressing the *CKX2* gene.** The table represents the complete data set, which is supplementary to Table 1. The table shows the mean cytokinin content in 1 g of extracted tissue in pmol/g F.W. ± SD (*n* = 3). Significance of differences compared to wild type (WT) was calculated using Student´s *t*-test (* p ≤ 0.05; ** p ≤ 0.01; *** p ≤ 0.001). LOD, limit of detection. NA, not applicable because two out of three biological replicates of this genotype have values below LOD.

| **Root 8-d-old** | |  |  |  |  |  |  |  |  |  |  |  |
| --- | --- | --- | --- | --- | --- | --- | --- | --- | --- | --- | --- | --- |
|  |  | **WT** |  | ***35S:CKX2-1*** | | | ***35S:CKX2-4*** | | | ***35S:CKX2-13*** | | |
|  |  | Mean | SD | Mean | SD | *t*-test to WT | Mean | SD | *t*-test to WT | Mean | SD | *t*-test to WT |
| iP-type CK |  |  |  |  |  |  |  |  |  |  |  |  |
|  | iP | 0.032 | 0.018 | <LOD | <LOD | <LOD | <LOD | <LOD | <LOD | 0.079 | 0.086 | - |
|  | iPR | 0.864 | 0.507 | <LOD | <LOD | <LOD | <LOD | <LOD | <LOD | 0.698 | 0.391 | - |
|  | iP9G | 0.261 | 0.009 | 0.060 | 0.006 | *** | 0.051 | 0.004 | *** | 0.036 | 0.005 | *** |
|  | iPR5´MP | 2.010 | 1.170 | 3.260 | 0.334 | - | 2.002 | 0.782 | - | 2.020 | 0.606 | - |
| *t*Z-type CK |  |  |  |  |  |  |  |  |  |  |  |  |
|  | *t*Z | 0.681 | 0.165 | 0.213 | 0.035 | ** | 0.213 | 0.106 | ** | 0.254 | 0.130 | * |
|  | *t*ZR | 0.094 | 0.003 | 0.047 | 0.002 | *** | 0.078 | 0.007 | ** | 0.074 | 0.022 | - |
|  | *t*Z9G | 0.296 | 0.035 | 0.205 | 0.117 | - | 0.082 | 0.016 | *** | 0.081 | 0.021 | *** |
|  | *t*ZOG | <LOD | <LOD | 0.212 | 0.136 | NA | 0.101 | NA | NA | 0.062 | NA | NA |
|  | *t*ZR5'MP | 0.317 | 0.077 | 0.248 | 0.008 | - | 0.345 | 0.095 | - | 0.241 | 0.160 | - |
| *c*Z-type CK |  |  |  |  |  |  |  |  |  |  |  |  |
|  | *c*Z | 0.232 | 0.048 | 0.089 | 0.010 | ** | 0.070 | 0.022 | ** | 0.059 | 0.020 | ** |
|  | *c*ZR | 1.376 | 0.384 | 1.599 | 0.558 | - | 1.495 | 0.238 | - | 1.269 | 0.512 | - |
|  | *c*Z9G | 2.382 | 0.160 | 2.141 | 0.174 | - | 1.803 | 0.122 | ** | 1.878 | 0.111 | ** |
|  | *c*ZOG | 0.211 | 0.046 | 0.127 | 0.025 | * | 0.149 | 0.034 | - | 0.322 | 0.185 | - |
|  | *c*ZR5´MP | 5.393 | 2.863 | 15.650 | 4.657 | * | 10.338 | 2.566 | * | 25.590 | 7.914 | * |

| **Shoot 8-d-old** | |  |  |  |  |  |  |  |  |  |  |  |
| --- | --- | --- | --- | --- | --- | --- | --- | --- | --- | --- | --- | --- |
|  |  | **WT** |  | ***35S:CKX2-1*** | | | ***35S:CKX2-4*** | | | ***35S:CKX2-13*** | | |
|  |  | Mean | SD | Mean | SD | *t*-test to WT | Mean | SD | *t*-test to WT | Mean | SD | *t*-test to WT |
| iP-type CK |  |  |  |  |  |  |  |  |  |  |  |  |
|  | iP | 0.035 | 0.008 | 0.066 | 0.037 | - | 0.060 | 0.037 | - | 0.013 | NA | NA |
|  | iPR | 0.467 | 0.311 | 1.124 | NA | NA | 0.387 | 0.232 | - | 0.843 | 0.457 | - |
|  | iP9G | 0.052 | 0.027 | 0.032 | NA | NA | <LOD | <LOD | <LOD | <LOD | <LOD | <LOD |
|  | iPR5´MP | 0.683 | 0.031 | 0.608 | 0.147 | - | 1.095 | 0.468 | - | 1.224 | 0.338 | - |
| *t*Z-type CK |  |  |  |  |  |  |  |  |  |  |  |  |
|  | *t*Z | 0.419 | 0.178 | <LOD | - | NA | <LOD | - | NA | 0.094 | 0.033 | * |
|  | *t*ZR | 0.053 | 0.012 | 0.022 | 0.002 | ** | 0.018 | 0.006 | ** | 0.031 | 0.009 | * |
|  | *t*Z9G | 0.433 | 0.198 | <LOD | <LOD | <LOD | 0.017 | NA | NA | <LOD | <LOD | <LOD |
|  | *t*ZOG | 0.805 | 0.225 | 0.266 | 0.180 | * | 0.225 | 0.118 | * | <LOD | <LOD | <LOD |
|  | *t*ZR5'MP | 0.323 | 0.118 | 0.234 | 0.117 | - | 0.072 | 0.017 | ** | 0.067 | 0.027 | ** |
| *c*Z-type CK |  |  |  |  |  |  |  |  |  |  |  |  |
|  | *c*Z | 0.053 | 0.017 | 0.033 | 0.017 | - | 0.018 | 0.005 | * | 0.018 | 0.008 | * |
|  | *c*ZR | 0.664 | 0.088 | 0.607 | 0.105 | - | 0.625 | 0.095 | - | 0.698 | 0.202 | - |
|  | *c*Z9G | 0.230 | 0.085 | 0.214 | 0.016 | - | 0.198 | 0.080 | - | 0.189 | 0.015 | - |
|  | *c*ZOG | <LOD | <LOD | <LOD | <LOD | <LOD | <LOD | <LOD | <LOD | 0.125 | 0.053 | NA |
|  | *c*ZR5´MP | 1.447 | 0.867 | 0.818 | NA | NA | 1.352 | NA | NA | 1.545 | NA | NA |

| **Seedlings 8-d-old** | |  |  |  |  |  |  |  |  |  |  |  |
| --- | --- | --- | --- | --- | --- | --- | --- | --- | --- | --- | --- | --- |
|  |  | **WT** | | ***35S:CKX2-1*** | | | ***35S:CKX2-4*** | | | ***35S:CKX2-13*** | | |
|  |  | Mean | SD | Mean | SD | *t*-test to WT | Mean | SD | *t*-test to WT | Mean | SD | *t*-test to WT |
| iP-type CK |  |  |  |  |  |  |  |  |  |  |  |  |
|  | iP | 0.208 | 0.078 | 0.088 | 0.029 | * | 0.087 | 0.033 | * | 0.108 | 0.036 | - |
|  | iPR | 0.257 | 0.158 | <LOD | <LOD | <LOD | 0.717 | - | NA | 0.494 | - | NA |
|  | iP9G | 0.050 | 0.013 | 0.023 | 0.010 | * | <LOD | - | NA | 0.020 | 0.010 | * |
| *t*Z-type CK |  |  |  |  |  |  |  |  |  |  |  |  |
|  | *t*Z | 0.087 | 0.049 | 0.050 | - | NA | 0.121 | 0.069 | - | 0.039 | 0.007 | * |
|  | *t*ZR | 0.090 | 0.016 | 0.029 | 0.006 | ** | 0.041 | 0.008 | ** | 0.028 | 0.011 | ** |
|  | *t*Z9G | 0.244 | 0.158 | 0.024 | - | NA | 0.069 | 0.037 | - | 0.018 | - | NA |
|  | *t*ZOG | 0.286 | 0.170 | 0.114 | - | NA | 0.078 | - | NA | <LOD | <LOD | <LOD |
|  | *t*ZR5'MP | 0.371 | 0.062 | 0.081 | 0.047 | ** | 0.086 | 0.045 | ** | 0.109 | 0.039 | ** |
| *c*Z-type CK |  |  |  |  |  |  |  |  |  |  |  |  |
|  | *c*Z | 0.072 | 0.010 | 0.037 | 0.021 | * | 0.041 | 0.017 | * | 0.040 | 0.019 | * |
|  | *c*ZR | 0.766 | 0.092 | 0.453 | 0.179 | * | 0.668 | 0.277 | - | 0.756 | 0.227 | - |
|  | *c*Z9G | 0.441 | 0.102 | 0.546 | 0.139 | - | 0.700 | 0.109 | * | 0.433 | 0.039 | - |
|  | *c*ZOG | 0.101 | 0.043 | 0.090 | - | NA | <LOD | <LOD | <LOD | <LOD | <LOD | <LOD |
|  | *c*ZR5´MP | 2.730 | - | 2.610 | 1.645 | NA | 4.722 | - | NA | 2.904 | 1.260 | - |

| **Seedlings 14-d-old** | |  |  |  |  |  |  |  |  |  |  |  |
| --- | --- | --- | --- | --- | --- | --- | --- | --- | --- | --- | --- | --- |
|  |  | **WT** | | ***35S:CKX2-1*** | | | ***35S:CKX2-4*** | | | ***35S:CKX2-13*** | | |
|  |  | Mean | SD | Mean | SD | *t*-test to WT | Mean | SD | *t*-test to WT | Mean | SD | *t*-test to WT |
| iP-type CK |  |  |  |  |  |  |  |  |  |  |  |  |
|  | iP | <LOD | <LOD | <LOD | <LOD | <LOD | <LOD | <LOD | <LOD | 0.029 | - | NA |
|  | iPR | 0.030 | 0.018 | <LOD | <LOD | <LOD | 0.019 | 0.011 | - | 0.027 | - | NA |
|  | iP9G | 0.848 | 0.509 | 0.777 | 0.213 | - | 0.691 | 0.272 | - | 0.911 | 0.506 | - |
|  | iPR5´MP | 0.000 | 0.000 | 0.000 | 0.000 | 0.000 | 0.000 | 0.000 | 0.000 | 0.000 | 0.000 | 0.000 |
| *t*Z-type CK |  |  |  |  |  |  |  |  |  |  |  |  |
|  | *t*Z | 0.261 | 0.050 | 0.029 | 0.010 | ** | 0.042 | 0.011 | *** | 0.047 | 0.010 | ** |
|  | *t*ZR | 0.489 | 0.143 | <LOD | <LOD | <LOD | <LOD | <LOD | <LOD | <LOD | <LOD | <LOD |
|  | *t*Z9G | 2.458 | 0.393 | 0.494 | - | NA | <LOD | <LOD | <LOD | <LOD | <LOD | <LOD |
|  | *t*ZOG | 1.334 | 0.358 | 0.102 | 0.037 | ** | 0.052 | 0.017 | ** | 0.172 | 0.046 | ** |
|  | *t*ZR5'MP | 0.089 | 0.017 | 0.088 | 0.018 | - | 0.090 | 0.007 | - | 0.086 | 0.007 | - |
| *c*Z-type CK |  |  |  |  |  |  |  |  |  |  |  |  |
|  | *c*Z | 0.420 | 0.149 | 0.399 | 0.153 | - | 0.491 | 0.181 | - | 0.381 | 0.136 | - |
|  | *c*ZR | 0.043 | 0.008 | 0.055 | 0.021 | - | 0.049 | 0.013 | - | 0.057 | 0.006 | * |
|  | *c*Z9G | 0.111 | 0.019 | 0.093 | 0.048 | - | 0.070 | 0.036 | * | 0.134 | 0.025 | - |
|  | *c*ZOG | 1.245 | 0.643 | <LOD | <LOD | <LOD | <LOD | <LOD | <LOD | 1.444 | - | NA |
|  | *c*ZR5´MP | 0.000 | 0.000 | 0.000 | 0.000 | 0.000 | 0.000 | 0.000 | 0 | 0.000 | 0.000 | 0.000 |
