# Supplementary material for: Root enhancement in cytokinin-deficient oilseed rape causes leaf mineral enrichment, increases the chlorophyll concentration under nutrient limitation and enhances the phytoremediation capacity
Source: BMC Plant Biol. 2019 Feb 20;19:83. doi: 10.1186/s12870-019-1657-6 (PMC6381662; doi:10.1186/s12870-019-1657-6)
Supplement: Supplementary file 2 — Table S2. Leaf element concentration in leaves of one-month-old Brassica napus plants overexpressing the CKX2 gene. The table represents the complete data set, which is supplementary to Fig. 5. The table shows the mean element content in 1 g of extracted tissue in mg g− 1 DW or μg g− 1 DW ± SD (n = 4 for each data point). Each biological replicates contains pooled leaf samples of two independent plants. Data were compared using Student’s t-test (* p ≤ 0.05; ** p ≤ 0.01; *** p ≤ 0.001). DW, dry weight. (DOC 46 kb) [file 12870_2019_1657_MOESM2_ESM.doc]

**Table S2 Leaf element content in leaves of one-month-old *Brassica napus* plants overexpressing the *CKX2* gene.** The table represents the complete data set, which is supplementary to Fig. 5. The table shows the mean element content in 1 g of extracted tissue in mg/g DW or µg/g DW ± SD (n = 4 for each data point). Each biological replicates contains pooled leaf samples of two independent plants. Data were compared using Student´s *t*-test (* p ≤ 0.05; ** p ≤ 0.01; *** p ≤ 0.001). DW, dry weight.

| Element concentration | **WT** | | ***35S:CKX2-4*** | | | ***35S:CKX2-13*** | | |
| --- | --- | --- | --- | --- | --- | --- | --- | --- |
|  | Mean | SD | Mean | SD | *t*-test to WT | Mean | SD | *t*-test to WT |
| B (µg/g DW) | 32.72 | 1.23 | 37.34 | 1.58 | ** | 40.32 | 1.34 | *** |
| Ca (mg/g DW) | 22.61 | 3.84 | 31.98 | 3.15 | ** | 35.17 | 2.75 | ** |
| Co (µg/g DW) | 4.35 | 0.15 | 4.64 | 0.26 | - | 4.73 | 0.18 | * |
| Cu (µg/g DW) | 7.95 | 0.55 | 10.28 | 0.50 | *** | 10.21 | 0.67 | ** |
| Fe (µg/g DW) | 88.97 | 44.62 | 73.22 | 5.13 | - | 79.43 | 3.62 | - |
| K (mg/g DW) | 35.94 | 2.51 | 38.92 | 3.09 | - | 43.36 | 1.30 | ** |
| Mg (mg/g DW) | 2.67 | 0.06 | 3.44 | 0.05 | *** | 3.53 | 0.15 | *** |
| Mn (µg/g DW) | 45.37 | 3.14 | 52.12 | 1.53 | * | 54.50 | 1.89 | ** |
| Mo (µg/g DW) | 1.38 | 0.12 | 2.41 | 0.18 | *** | 3.17 | 0.09 | *** |
| Na (mg/g DW) | 6.35 | 1.35 | 7.24 | 1.40 | - | 7.84 | 1.37 | - |
| P (mg/g DW) | 6.94 | 0.40 | 7.85 | 0.73 | - | 8.04 | 0.62 | * |
| S (mg/g DW) | 11.42 | 1.11 | 16.17 | 0.97 | *** | 19.95 | 0.46 | *** |
| Zn (µg/g DW) | 39.77 | 2.41 | 50.26 | 1.76 | *** | 52.36 | 0.91 | *** |
